# Construction of a nomogram for predicting the risk of all-cause mortality in patients with diabetic retinopathy

**DOI:** 10.3389/fendo.2025.1493984

**Published:** 2025-02-21

**Authors:** Wenwei Zuo, Xuelian Yang

**Affiliations:** ^1^ School of Gongli Hospital Medical Technology, University of Shanghai for Science and Technology, Shanghai, China; ^2^ Department of Neurology, Shanghai Pudong New Area Gongli Hospital, Shanghai, China

**Keywords:** diabetic retinopathy, all-cause mortality, NHANES, nomogram, type 2 diabetes

## Abstract

**Background:**

Diabetic retinopathy (DR) not only leads to visual impairment but also increases the risk of death in type 2 diabetes patients. This study aimed to construct a nomogram to assess the risk of all-cause mortality in patients with DR.

**Methods:**

This cross-sectional study included 1004 patients from the National Health and Nutrition Examination Survey database (NHANES) between 1999-2018. Participants were randomized in a 7:3 ratio into a training set and a test set. We selected predictors by LASSO regression and multifactorial Cox proportional risk regression analysis and constructed nomograms, guided by established clinical guidelines and expert consensus as the gold standard. We used the concordance index (C-index), receiver operating characteristic curve (ROC), calibration curve, and decision curve analysis (DCA) to evaluate the nomogram’s discriminative power, calibration quality, and clinical use.

**Results:**

The training and test sets consisted of 703 and 301 participants with a median age of 64 and 63 years, respectively. The study identified seven predictors, including age, marital status, congestive heart failure (CHF), coronary heart disease (CHD), stroke, creatinine level, and taking insulin. The C-index of the nomogram model constructed from the training set was 0.738 (95% CI: 0.704-0.771), while the C-index of the test set was 0.716 (95% CI: 0.663-0.768). In the training set, the model’s AUC values for predicting all-cause mortality risk at 3 years, 5 years, and 10 years were 0.739, 0.765, and 0.808, respectively. In the test set, these AUC values were 0.737, 0.717, and 0.732, respectively. The ROC curve, calibration curve, and DCA curve all demonstrated excellent predictive performance, confirming the model’s effectiveness and reliability in clinical applications.

**Conclusions:**

Our nomogram demonstrates high clinical predictive accuracy, enabling clinicians to effectively predict the overall mortality risk in patients with DR, thereby significantly improving their prognosis.

## 1 Introduction

Diabetic retinopathy (DR) is a prevalent and severe microvascular complication among individuals with diabetes, representing a significant public health challenge globally (1). A meta-analysis indicates that approximately 22.27% of people with diabetes worldwide have DR, with projections suggesting this number will reach 160.5 million by 2045 (2). In the United States, studies based on nationally representative data estimate that approximately 9.6 million patients (95% uncertainty interval [UI]: 7.90-11.55) were affected by DR in 2021 (3). The primary characteristics of DR include retinal vascular damage, which often results in vascular leakage, neovascularization, and retinal dysfunction (4). As the disease advances, patients experience progressive visual impairment, severely impacting their quality of life (5). Given that the retina is a microvascular structure highly susceptible to changes in blood flow and composition, chronic systemic diseases can significantly damage retinal integrity (6–8). This not only threatens patients’ vision but also increases the likelihood of broader health issues, underscoring the importance of early detection and intervention.

Moreover, DR is not only a cause of visual impairment but also significantly correlates with the overall health of patients, particularly increasing their mortality risk. Research showed that mortality in diabetic patients was strongly linked to the presence and severity of DR (9). Epidemiological studies revealed that diabetic patients with retinopathy had a notably higher mortality rate compared to those without. The elevated mortality risk was attributable to the development of cardiovascular disease, renal impairment, and other diabetes-related complications (10–12). Patients with DR have an increased likelihood of cardiovascular events, and following such events, their mortality risk escalates substantially (13). Therefore, early recognition of DR is critical not only for preserving vision but also as an important indicator of a patient’s general health. Despite the well-established association between DR and mortality risk, there is a lack of predictive models or tools specifically designed to assess mortality risk in this patient group. Traditional mortality risk prediction tools typically focus on assessing the risk for cardiovascular disease or other single conditions, failing to adequately consider the unique clinical characteristics and combined risks faced by patients with DR. This gap in predictive tools limits clinicians’ ability to develop individualized treatment plans.

To address this problem, a straightforward, useful, and precise nomogram for estimating the risk of all-cause mortality in patients with DR was developed and validated using data from the National Health and Nutrition Examination Survey (NHANES) from 1999 to 2018. This nomogram is designed to help clinicians identify high-risk patients early and implement proactive interventions. Additionally, it aims to increase awareness among patients and their families, promoting proactive health management.

## 2 Methods

### 2.1 study design and study population

The National Health and Nutrition Examination Survey (NHANES) is a cross-sectional survey conducted every two years in the United States that was created in 1999 to evaluate the nutritional status and general health of non-institutionalized citizens. To guarantee national representation, a stratified, intricate, multistage, and probabilistic sampling methodology is employed in the survey. The sampling method for this survey specifically involves stratified sampling. Surveys are conducted biennially, beginning with the selection of several states across the United States. Within these selected states, specific counties are chosen, followed by the selection of municipalities within those counties. Finally, households within the selected municipalities are identified as the units of analysis for the survey (14, 15). All participants gave their informed agreement, and the National Center for Health Statistics (NCHS) Institutional Review Board approved the study procedure. Data for this cross-sectional study were drawn from NHANES between 1999 and 2018.

In this study, we collected data on patients diagnosed with diabetic retinopathy over 10 periods from 1999 to 2018, totaling 1500 individuals. To enhance data integrity and analytical accuracy, we applied the following exclusion criteria: first, we excluded 3 pregnant women. Second, we excluded 493 individuals due to missing data on key covariates, which prevented comprehensive analysis. After these screening steps, the final sample size included in the analysis was 1004 individuals.

### 2.2 Determination of diabetic retinopathy

DR status was determined through participant responses to the following questions: “Have you ever been told by your doctor that diabetes is affecting your eyes?” and “Have you ever been diagnosed with retinopathy?”. This approach was based on previously published literature, which supported its reliability in assessing the status of patients with diabetic retinopathy (16–19).

### 2.3 Determination of mortality status

The NHANES Public Use Linked Mortality File, which combines NHANES data with death certificate information from the National Death Index, was the source of the mortality data. The file was updated as of December 31, 2019 (20). Every participant’s follow-up period was determined by taking the date of their NHANES enrollment and adding the date of their death or the follow-up period’s expiration.

### 2.4 Predictors

Variable selection was conducted based on previous literature and clinical characteristics (21–23), we collected the following potential characteristics: demographic information including age, sex (male and female), race (Mexican American, other Hispanic, non-Hispanic white, non-Hispanic black, and other races), education level (less than high school, high school or equivalent, and college and above), marital status (married/living with a partner, widowed/divorced/separated, and never married), and poverty income ratio (PIR). For physical examination, we recorded body mass index (BMI). For lifestyle, smoking history (having smoked at least 100 cigarettes in a lifetime), drinking (having consumed at least 12 drinks of any type of alcoholic beverage in any given year), and physical activity (engaging in vigorous activity for at least 10 minutes, which leads to significant sweating or a marked increase in breathing rate and heart rate) were assessed. For disease history, we looked at hypertension, congestive heart failure (CHF), coronary heart disease (CHD), stroke, and cancer, all of which were obtained by patient self-report or physician diagnosis. Relevant disease information can be found at https://wwwn.cdc.gov/nchs/nhanes/search/default.aspx. In addition, we obtained information on whether patients used insulin and other diabetes medications through questionnaires, as well as total cholesterol, high-density lipoprotein cholesterol, glycosylated hemoglobin, creatinine, white blood cells, lymphocytes, monocytes, red blood cells, and platelets from laboratory tests.

### 2.5 Construction model

The dataset was divided 7:3 into training and test sets. The most important predictors in the training set were found using the least absolute shrinkage and selection operator (LASSO) regression model. These predictors were then used in a Cox proportional hazards regression analysis to identify independent predictors, with significance defined as a p-value of less than 0.05. The hazard ratio (HR) and their 95% confidence intervals were calculated. A nomogram was subsequently constructed using the identified predictors.

### 2.6 Model evaluation

The accuracy and performance of the risk prediction models were evaluated using several metrics. Receiver Operating Characteristic (ROC) curves were employed to assess model performance over time, with the area under the ROC curve (AUC) used as an indicator of accuracy; values closer to 1 represent better predictive performance (24). The concordance index (C-Index) was used to evaluate the discrimination performance of the model in more detail. A C-index of 0.7 or greater was deemed to represent good discrimination (25). The exact calculation of the C-Index is described in the **Supplementary Material** provided. Calibration curves were utilized to evaluate the model’s calibration, with an ideal 45-degree line indicating that observed probabilities matched expected probabilities. Lastly, Decision Curve Analysis (DCA) is a method used to evaluate the practical value of predictive models in clinical decision-making. This analysis achieves its purpose by comparing the net benefits of different decision strategies within a specific range of decision thresholds. Here, “net benefit” refers to the net effect after considering the benefits and losses associated with false positives and false negatives. The core principle of DCA is to quantify the utility of predictive models at specific clinical decision thresholds and compare it with other decision strategies, such as treating all patients or treating none. This method provides clinicians with an effective tool to select the optimal treatment approach in actual decision-making processes (26).

### 2.7 Statistical analysis

The interquartile range (IQR) and median were used to summarize non-normally distributed continuous variables, and percentages and frequencies were used to describe categorical variables. The Mann-Whitney U test was used to assess group differences for non-normally distributed continuous data, while the chi-square test was used to analyze differences in categorical variables.

R software version 4.3.2 was used for all statistical analyses, and a p-value of less than 0.05 was deemed statistically significant.

## 3 Results

### 3.1 Baseline characteristics

**Figure 1:**
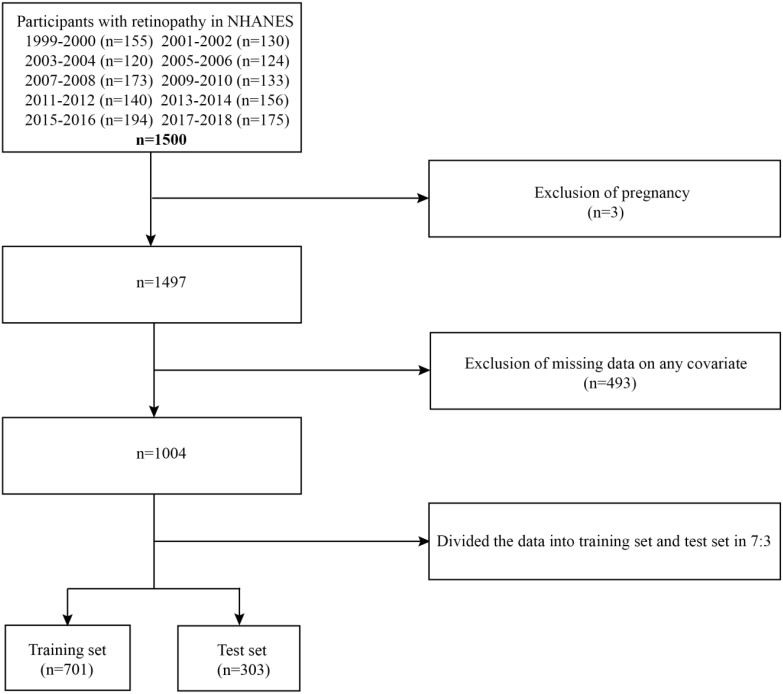
Flowchart of training and test sets.

This study included 1004 participants in total (**Figure 1**), with a median age of 63 years. Of these, 456 (45.42%) were women and 548 (54.58%) were men. A median follow-up length of 79 months was observed, during which 354 deaths among DR patients took place. We divided the dataset in a 7:3 ratio between a training set (701 participants) and a test set (303 participants) to achieve a balanced division. Participants in the training set had a median age of 64 years, with 389 (55.49%) men and 312 (44.51%) women. The test set had a median age of 63 years, with 159 (52.48%) males and 144 (47.52%) females. There were statistically significant variations between the two groups in terms of lymphocyte count, platelet count, PIR, cancer, and marital status (P<0.05). Nevertheless, no statistically significant variations were observed in other broad clinical characteristics or laboratory test results (**Table 1**).

**Table 1:** Baseline characteristics of patients in the training and test sets.

Characteristic	Overall	Training set	Test set	p-value
N	1004	701	303	
Age, (years)	63.00 (54.00-71.00)	64.00 (55.00-71.00)	63.00 (52.50-70.00)	0.076
Sex				0.378
Male	548 (54.58%)	389 (55.49%)	159 (52.48%)	
Female	456 (45.42%)	312 (44.51%)	144 (47.52%)	
Race				0.878
Mexican American	196 (19.53%)	133 (18.97%)	63 (20.79%)	
Other Hispanic	99 (9.86%)	73 (10.41%)	26 (8.58%)	
Non-Hispanic White	352 (35.06%)	247 (35.24%)	105 (34.65%)	
Non-Hispanic Black	265 (26.39%)	183 (26.11%)	82 (27.06%)	
Other Race	92 (9.16%)	65 (9.27%)	27 (8.91%)	
Education level				0.942
Less than high school	212 (21.11%)	149 (21.26%)	63 (20.79%)	
High school or equivalent	406 (40.44%)	281 (40.09%)	125 (41.25%)	
College or above	386 (38.45%)	271 (38.66%)	115 (37.95%)	
Marital status				0.019
Married/Living with Partner	597 (59.46%)	434 (61.91%)	163 (53.80%)	
Widowed/Divorced/Separated	335 (33.37%)	225 (32.10%)	110 (36.30%)	
Never married	72 (7.17%)	42 (5.99%)	30 (9.90%)	
PIR	1.56 (0.96-2.90)	1.65 (0.99-3.08)	1.43 (0.90-2.49)	0.021
BMI	30.90(26.60-36.70)	30.90 (26.77-36.50)	30.80 (26.47-37.04)	0.927
Smoking				0.993
No	484 (48.21%)	338 (48.22%)	146 (48.18%)	
Yes	520 (51.79%)	363 (51.78%)	157 (51.82%)	
Drinking				0.481
No	368 (36.65%)	252 (35.95%)	116 (38.28%)	
Yes	636 (63.35%)	449 (64.05%)	187 (61.72%)	
Hypertension				0.633
No	272 (27.09%)	193 (27.53%)	79 (26.07%)	
Yes	732 (72.91%)	508 (72.47%)	224 (73.93%)	
CHF				0.704
No	845 (84.16%)	592 (84.45%)	253 (83.50%)	
Yes	159 (15.84%)	109 (15.55%)	50 (16.50%)	
CHD				0.348
No	845 (84.16%)	585 (83.45%)	260 (85.81%)	
Yes	159 (15.84%)	116 (16.55%)	43 (14.19%)	
Stroke				0.541
No	868 (86.45%)	603 (86.02%)	265 (87.46%)	
Yes	136 (13.55%)	98 (13.98%)	38 (12.54%)	
Cancer				0.041
No	875 (87.15%)	601 (85.73%)	274 (90.43%)	
Yes	129 (12.85%)	100 (14.27%)	29 (9.57%)	
Physical activity				0.782
No	914 (91.04%)	601 (90.65%)	254 (90.07%)	
Yes	90 (8.96%)	62 (9.35%)	28 (9.93%)	
Taking insulin				0.522
No	528 (52.59%)	364 (51.93%)	164 (54.13%)	
Yes	476 (47.41%)	337 (48.07%)	139 (45.87%)	
Take diabetic pills				0.737
No	319 (31.77%)	225 (32.10%)	94 (31.02%)	
Yes	685 (68.23%)	476 (67.90%)	209 (68.98%)	
TC, (mmol/L)	4.60 (3.90-5.53)	4.55 (3.88-5.48)	4.71 (3.96-5.66)	0.079
HDL-C, (mmol/L)	1.19 (0.98-1.42)	1.16 (0.98-1.42)	1.19 (0.99-1.45)	0.986
HbA1c (%)	7.30 (6.50-8.77)	7.30 (6.40-8.80)	7.30 (6.50-8.60)	0.683
Creatinine	83.10 (66.30-114.92)	83.98 (67.18-114.04)	82.21 (63.65-115.36)	0.418
WBC, 10^3^/uL	7.40 (6.00-8.90)	7.30 (5.90-8.80)	7.60 (6.20-8.90)	0.125
Lymphocyte, 10^3^/uL	2.00 (1.50-2.60)	2.00 (1.50-2.60)	2.10 (1.60-2.80)	0.006
Monocyte, 10^3^/uL	0.60 (0.40-0.70)	0.50 (0.40-0.70)	0.60 (0.50-0.70)	0.247
Red blood cell, 10^6^/uL	4.55 (4.18-4.89)	4.54 (4.18-4.88)	4.56 (4.18-4.99)	0.266
Platelet, 10^3^/uL	234.50 (194.00-281.00)	233.00 (191.00-277.00)	238.00 (199.00-287.00)	0.040

PIR, poverty income ratio; BMI, body mass index; CHF, congestive heart failure; CHD, coronary heart disease; TC, total cholesterol; HDL-C, high-density lipoprotein cholesterol; WBC, White blood cell.

### 3.2 Constructing the model

**Figure 2:**
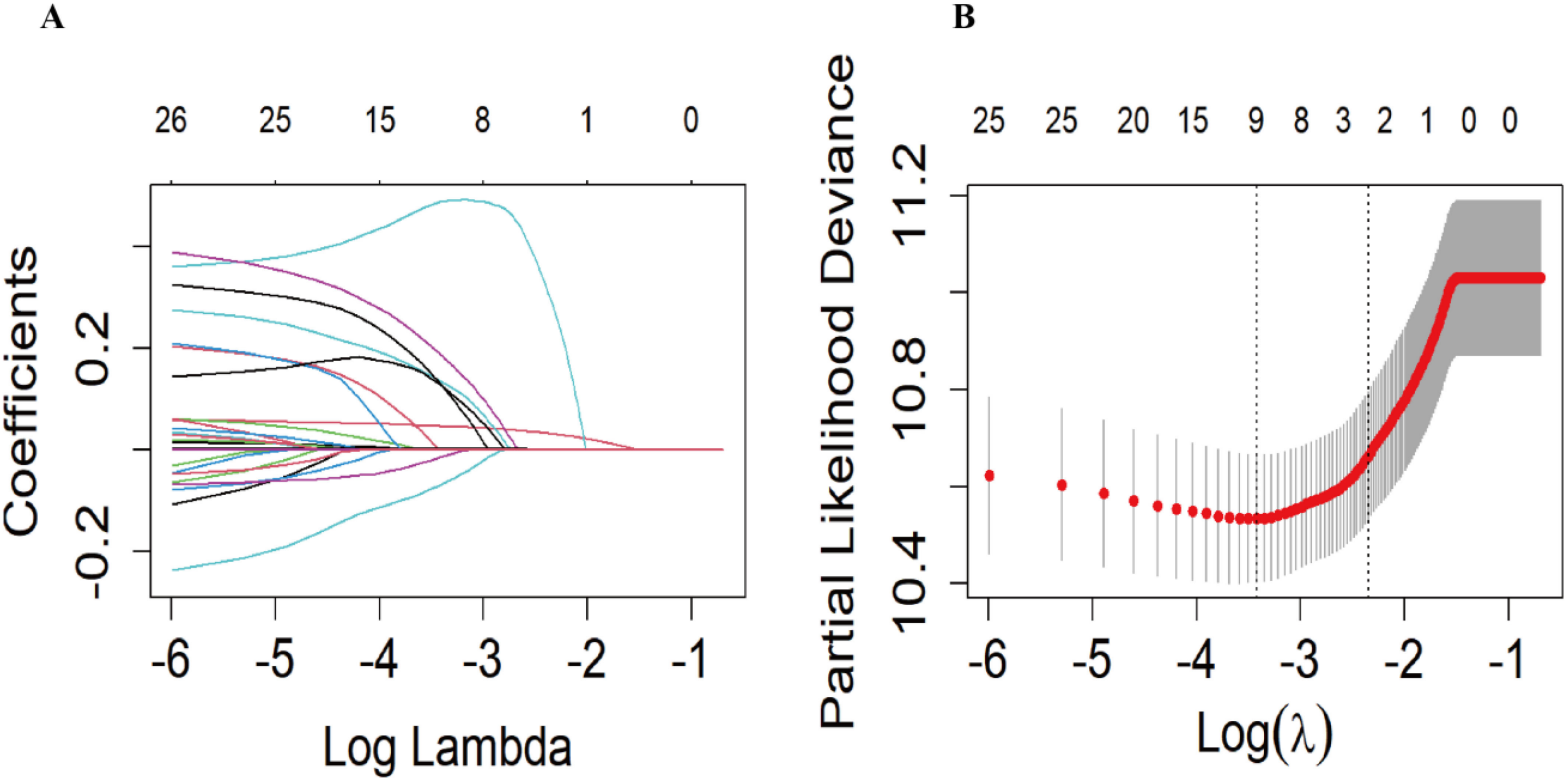
The Least absolute shrinkage and selection operator (LASSO) regression model was used to screen the predictors. **(A)** Generation of coefficient curves based on log(lambda) sequences with nonzero coefficients generated by optimal lambda. **(B)** The optimal parameters (lambda) in the LASSO model were selected by 10-fold cross-validation based on the minimum criterion. Vertical dashed lines are drawn on the optimal values based on the minimum mean square error (left side).

**Figure 3:**
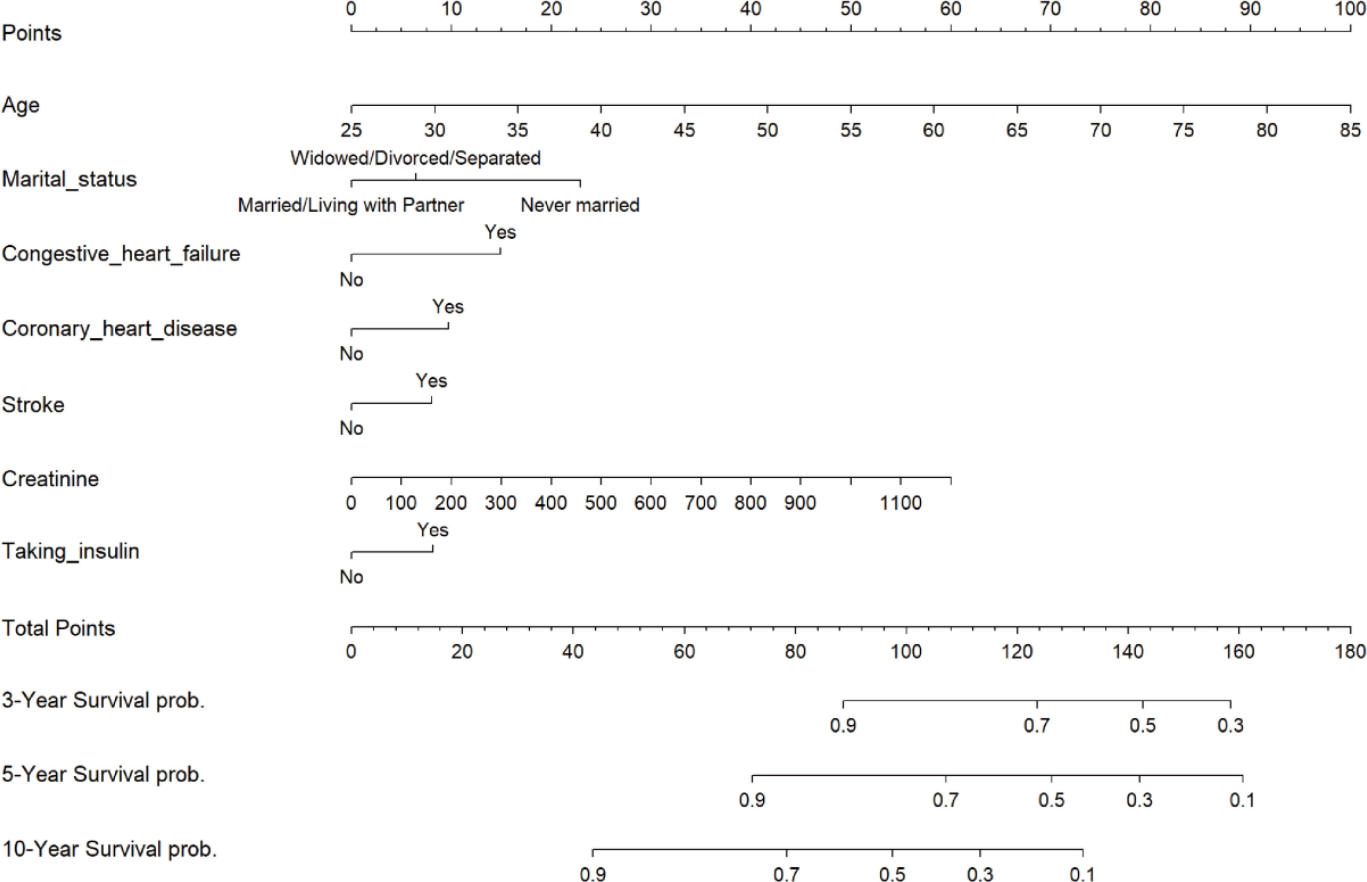
Nomogram of risk of all-cause mortality after 3, 5, and 10 years in patients with diabetic retinopathy. .

In the training set, we used the LASSO regression model to identify nine factors with non-zero coefficients, including age, marital status, PIR, CHF, CHD, stroke, taking insulin, red blood cell count, and creatinine levels (**Figure 2**). Using multivariable Cox proportional hazards regression analysis, seven of these variables were further found to be independent predictors of mortality risk in patients with DR. The independent predictors were: age (HR: 1.05, 95% CI: 1.04-1.07), marital status (divorced/widowed/separated HR: 1.13, 95% CI: 0.85-1.50 compared to married; never married HR: 2.06, 95% CI: 1.19-3.58), CHF (HR: 1.58, 95% CI: 1.12-2.24), CHD (HR: 1.45, 95% CI: 1.04-2.01), stroke (HR: 1.41, 95% CI: 1.01-1.98), taking insulin (HR: 1.31, 95% CI: 1.01-1.71), and creatinine levels (HR: 1.00, 95% CI: 1.00-1.00) (**Supplementary Table S1**). These seven independent predictors were then used to construct the nomogram (**Figure 3**).

### 3.3 Evaluation of the model

**Figure 4:**
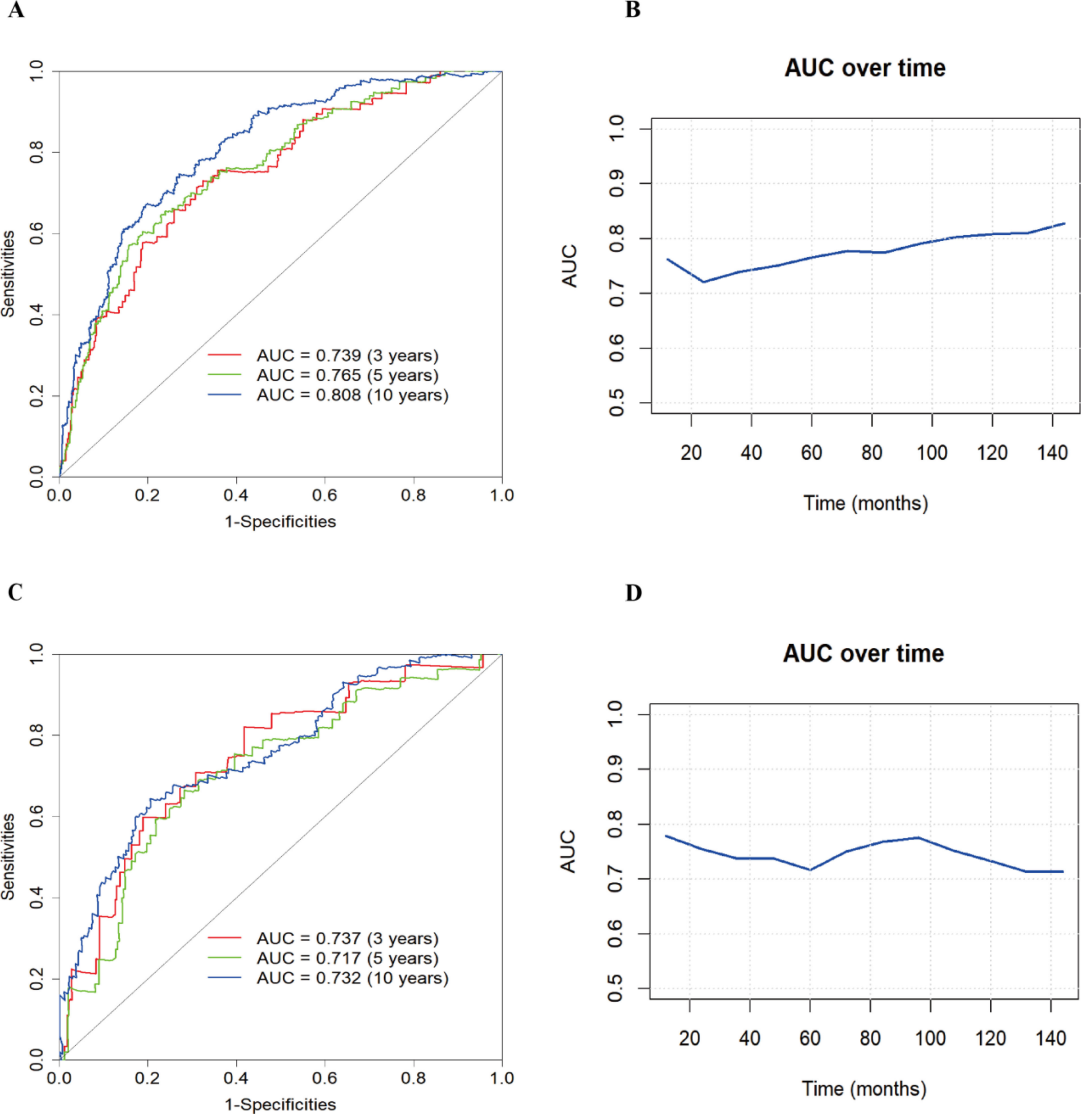
Nomogram modeling of all-cause mortality in patients with diabetic retinopathy for ROC analysis and AUC curves over time. **(A)** ROC curve of the training set; **(B)** AUC curve of the training set; **(C)** ROC curve of the test set; **(D)** AUC curve of the test set.

To evaluate the robustness of the constructed clinical prediction models, we tested them using both the training and test sets and calculated the overall C-index of the models. For the training set, the nomogram model’s C-index was 0.738 (95% CI: 0.704-0.771), and for the test set, it was 0.716 (95% CI: 0.663-0.768). Furthermore, a time-dependent receiver operating characteristic (ROC) curve analysis was conducted to evaluate the nomogram model’s prediction accuracy for the probability of all-cause mortality at various time intervals. The results showed that in the training set, the AUC values of the model for predicting 3-, 5-, and 10-year all-cause mortality risk were 0.739, 0.765, and 0.808, respectively, while in the test set, these values were 0.737, 0.717, and 0.732, respectively (**Figure 4**).

**Figure 5:**
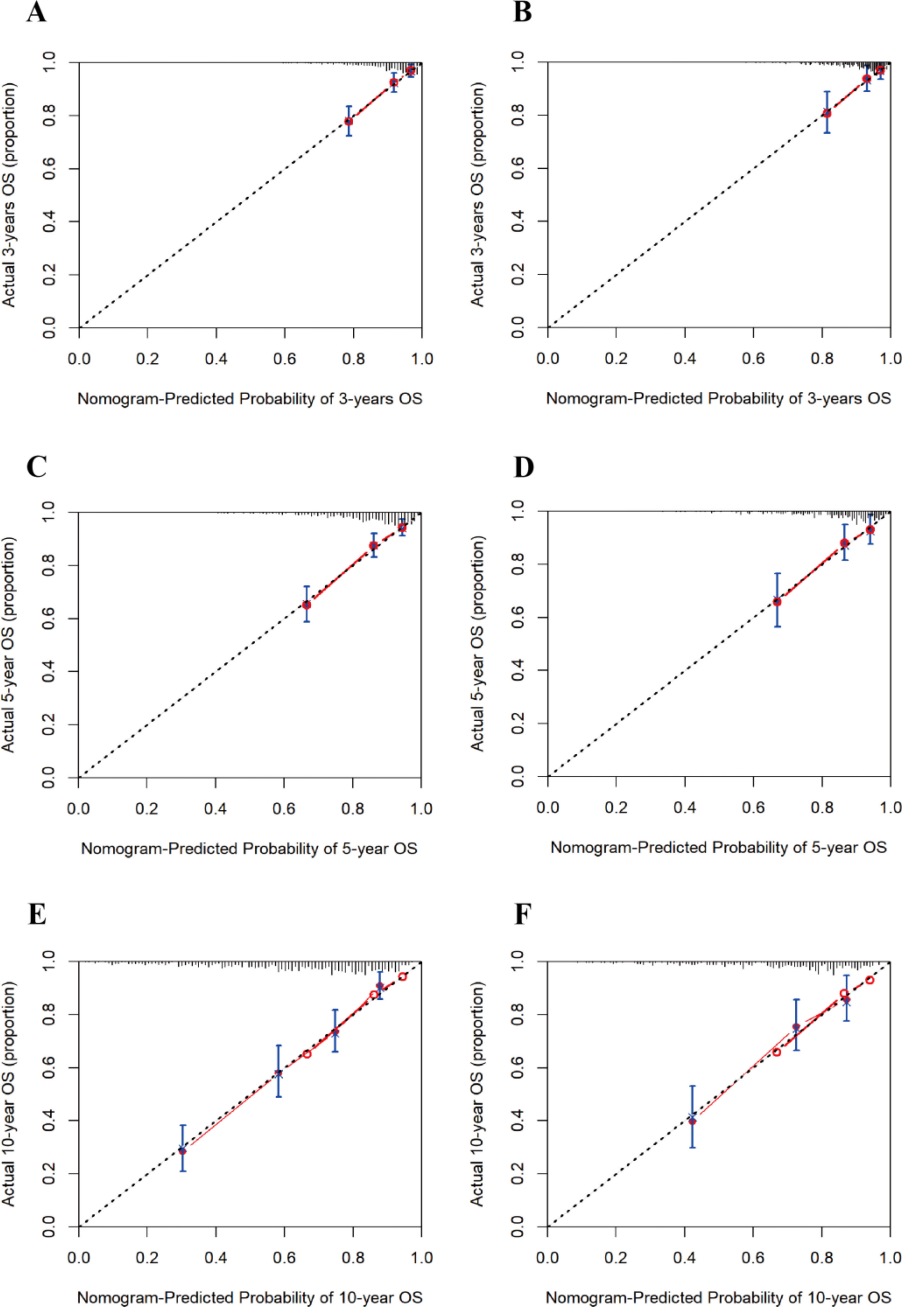
Calibration curves for 3-, 5-, and 10-year risk of all-cause mortality in patients with diabetic retinopathy. **(A)** 3-year training set; **(B)** 3-year test set; **(C)** 5-year training set; **(D)** 5-year test set; **(E)** 10-year training set; **(F)** 10-year test set.

The calibration curves of the nomogram model for both the training and test sets demonstrated that the predicted probabilities closely aligned with the actual observed probabilities, forming a relationship that roughly followed a 45-degree diagonal line. This indicates that the nomogram model has a good agreement between predicted and actual event probabilities (**Figure 5**).

**Figure 6:**
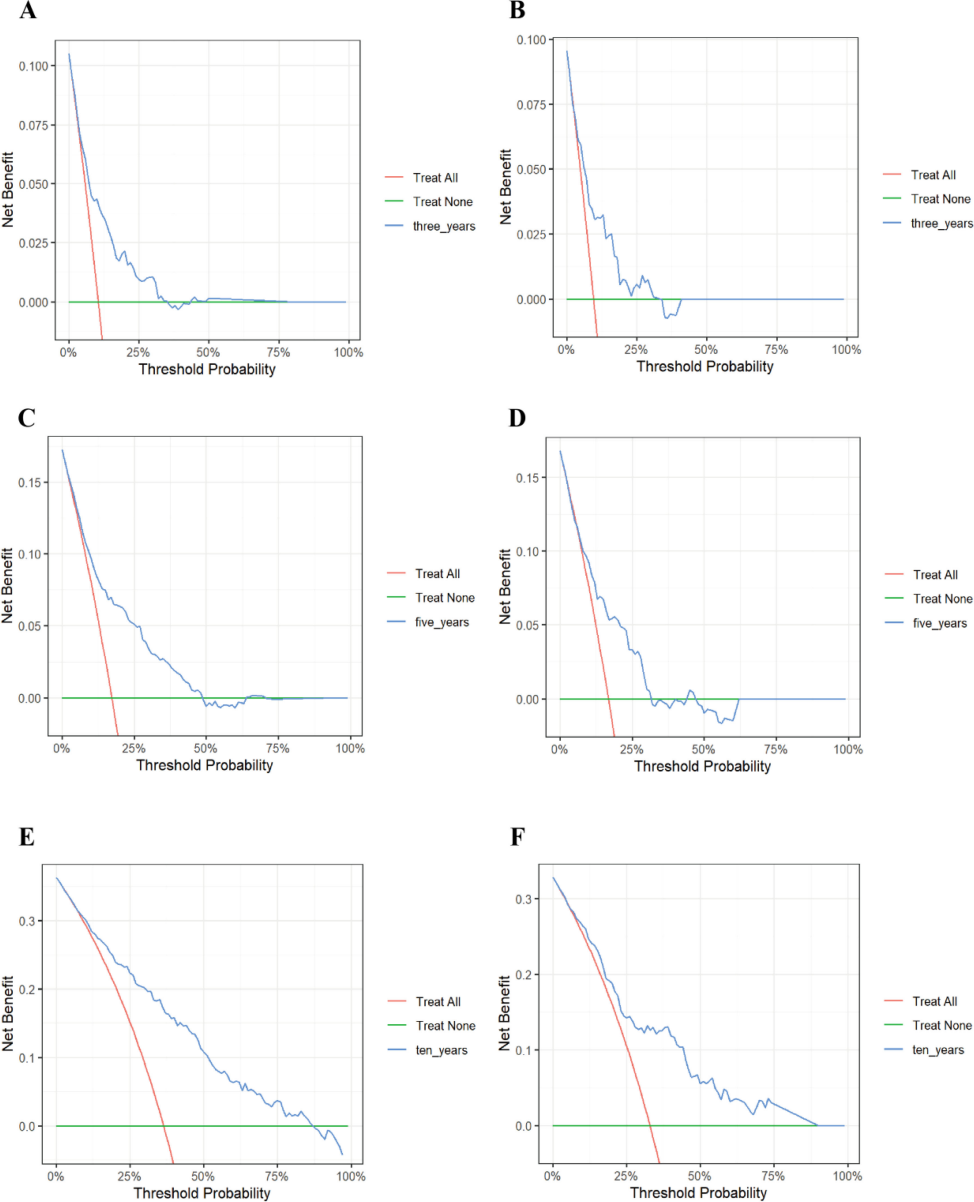
Decision curve analysis of the 3-, 5-, and 10-year risk of all-cause mortality in patients with diabetic retinopathy. **(A)** 3-year training set; **(B)** 3-year test set; **(C)** 5-year training set; **(D)** 5-year test set; **(E)** 10-year training set; **(F)** 10-year test set. The vertical coordinate represents the net benefit and the horizontal coordinate represents the critical probability. The red line represents “All” (assuming all participants receive the intervention), while the green line represents “None” (assuming all participants do not receive the intervention). The blue line is the DCA curve then reflects the net benefit at different decision thresholds. If the model consistently remains above the “All” line and the “None” line across a broad range of threshold values, this indicates that the model possesses practical applicability in various clinical contexts.

Furthermore, our research findings indicated that in decision analysis, the model curve was above the “All (all interventions)” line and the “None (no interventions)” line within the clinically relevant threshold range. This finding suggested that the model could provide significant net benefits in clinical applications, supporting its potential value in actual medical decision-making. Specifically, as the prediction time extended, we observed that the net benefits of the 10-year diabetes mortality risk prediction were significantly greater than those of the 3-year and 5-year predictions. This suggested that clinicians could use the model to make more informed decisions among multiple treatment options, thereby potentially improving patient prognosis and survival outcomes (**Figure 6**). Collectively, these results supported the clinical applicability of the nomogram model and provided a reliable foundation for patient risk assessment.

## 4 Discussion

To date, there are no satisfactory predictive tools that effectively identify the risk of all-cause mortality in patients with DR. To address this issue, we constructed a nomogram using the NHANES database from 1999 to 2018, aiming to accurately assess the likelihood of all-cause mortality in this specific population. The model included seven predictors: age, marital status, CHF, CHD, stroke, creatinine level, and taking insulin. The model showed excellent discriminative ability in both the training and test sets, with C-indexes of 0.738 and 0.716, respectively, demonstrating the great accuracy of the model in identifying the risk for mortality for various patients. Furthermore, the calibration curve analysis results showed that the model had very high accuracy in both groups, demonstrating a strong correlation between the model’s predictions and the actual observations. To further validate the clinical utility of the model, DCA was performed, which showed that the model provided good clinical decision support at different thresholds.

Our study found that the risk of death in patients with DR increased significantly with increasing age. Age is an important factor affecting the health status of diabetic patients, and higher age is usually accompanied by multiple comorbidities, which further aggravate the patient’s burden and increase the risk of death (27). Studies have shown that in older diabetic patients, the incidence of cardiovascular disease and nephropathy is significantly higher than in younger patients, leading to a significant increase in their all-cause mortality (28, 29). Additionally, the incidence of DR increased with age, which was closely related to the aging of retinal blood vessels and the long-term effects of diabetes on the microvasculature (30, 31). At the same time, older patients often face additional challenges in diabetes management, including drug interactions, comorbidity management, and decreased quality of life, all of which may lead to decreased treatment adherence and thus further increase the risk of death (32–34). Therefore, individualized management strategies for elderly patients with DR are particularly important to reduce their risk of death and improve their quality of life. Marital status has a significant effect on the risk of death in patients with DR. Studies have shown that married patients usually have a lower risk of death than unmarried patients, a phenomenon that may be closely related to various factors such as social support, mental health, and health behaviors (35). A study from the United States showed that divorce or separation status was significantly associated with diabetes mortality in men (HR: 1.318, 95% CI: 1.010-1.719; HR: 1.283, 95% CI: 1.054-1.562). For women, widowed status was similarly associated with diabetes mortality (HR: 1.349, 95% CI: 1.107-1.643; HR: 1.262, 95% CI: 1.113-1.431) (36). Married individuals usually enjoyed stronger social support networks and could receive support from their partners both emotionally and practically, which made them more likely to maintain a positive psychological state in the face of illness (37, 38). Thus, marital status may be considered an important social factor that influences survival in patients with DR. Future studies should further explore how social support can be utilized to improve health outcomes in patients with DR.

Patients with DR who have a history of comorbid CHF, CHD, and stroke face a higher risk of death. CHF is one of the common complications in patients with diabetes, and studies have shown that it is positively associated with all-cause mortality and cardiovascular mortality (39). Heart failure leads to cardiac decompensation, which affects systemic circulation, exacerbating the development of other diseases, including DR (40). Therefore, patients with heart failure have a significantly increased risk of death following a cardiovascular event. CHD is one of the most common cardiovascular diseases among diabetic patients, and studies have shown that the incidence of coronary artery disease is higher in diabetic patients than in non-diabetic patients (41). When CHD is combined with DR, the risk of cardiovascular events is significantly increased, leading to higher mortality rates (42). This phenomenon may be related to the impairment of vascular endothelial function and chronic inflammatory responses caused by diabetes, which accelerate atherosclerosis formation and ultimately lead to cardiovascular events (43, 44). In addition, a history of stroke is an important predictor of mortality risk in patients with DR. A prospective cohort study found that a history of clinical stroke significantly increased the risk of cardiovascular disease-related death in patients with retinopathy (HR: 3.30, 95% CI: 2.05-5.32) (45). Stroke not only leads to acute disability but also severely affects patients’ quality of life and self-care ability, increasing the difficulty of long-term care and health management, thus increasing mortality (46). Therefore, clinicians should pay great attention to these comorbidities and develop more effective intervention strategies in diabetes management to reduce the risk of patient mortality.

The present study showed a significant association between elevated creatinine levels and the risk of death in patients with DR. Creatinine is a waste product of muscle metabolism that is usually excreted by the kidneys, and elevated levels often indicate impaired renal function (47). In patients with diabetes, worsening kidney function not only signals the progression of diabetic nephropathy but may also be associated with systemic problems such as cardiovascular disease, chronic inflammation, and increased oxidative stress (48, 49). High creatinine levels usually reflect more severe diabetic complications, and the severity of retinopathy is also strongly associated with overall health (50). Studies have found that diabetics who also have high creatinine levels are at higher risk of death (51). This phenomenon may be related to the chronic inflammatory state and metabolic disorders triggered by high creatinine levels, which affect the patient’s overall health (52). Therefore, regular monitoring of kidney function and fundus health is crucial for diabetic patients. Early detection of retinopathy and renal function problems can help in taking effective interventions, thereby reducing the risk of death. In addition, our study found that immediate insulin administration was a significant predictor of mortality risk in patients with DR. DR is usually the result of chronic hyperglycemia in diabetic patients, whose overall health may have been severely compromised (53). A large controlled cohort study showed that glucose-lowering medications were associated with increased mortality in diabetic patients of all ages (54). The possible explanation is that although immediate insulin administration is effective in lowering blood glucose, it may not rapidly improve the general health of these patients and may instead trigger complications such as hypoglycemia, which can increase the risk of death (55, 56). Therefore, the manner and timing of glycemic control in patients with DR need to be considered in the context of their overall health status and potential risks.

Identifying predictors and performing high-risk stratification are important medical decisions for preventing death in patients with DR. To this end, this study included patients with DR from the NHANES database between 1999 and 2018, with all-cause mortality as an outcome indicator, to develop a validated clinical prediction model to assess the risk of all-cause mortality in these patients. We used Lasso regression for variable selection, a method that effectively identifies significant predictors associated with mortality risk and prevents overfitting by adjusting the complexity of model fitting, making the model more robust and plausible. Additionally, we applied Cox proportional risk regression analysis to further evaluate the screened predictors to determine their statistical significance in mortality risk prediction. We developed a nomogram model based on these screened variables, designed to transform complex regression equations into visual graphs that enable clinicians to understand and interpret the results of the prediction model more intuitively. With this model, we assign a score to each predictor and aggregate all scores to calculate the probability of a risk event for each patient. Clinicians can use this tool to identify high-risk patients promptly so that appropriate interventions can be implemented to improve patient survival. For example, consider a 60-year-old married patient with congestive heart failure, coronary artery disease, and stroke and a blood creatinine level of 200 µmol/L who is not taking insulin. According to our predictive model, this patient has a total score of approximately 100, and his or her 3-year survival rate is approximately 0.95, the 5-year survival rate is approximately 0.75, and the 10-year survival rate is approximately 0.45. Such survival data provide clinicians with important information that can help with more precise risk management and intervention planning.

There are several limitations to our study. First, the diagnosis of DR was based on self-reported information from the NHANES, which may introduce information bias due to cognitive deficits or recall bias in participants. Second, participants with missing data were usually in poorer health, which may have led to fewer observed deaths, thus introducing selection bias. Additionally, our model has only been internally validated, and external validation is yet to be conducted to ensure its reliability and generalizability. Finally, the sample in this study was limited to patients with DR in the United States, which may limit the applicability of the model to other countries and regions.

## 5 Conclusions

This study successfully developed, validated, and visualized a nomogram for predicting the risk of all-cause mortality in patients with DR at 3, 5, and 10 years. The nomogram incorporates seven common clinical characteristics: age, marital status, CHF, CHD, stroke, creatinine levels, and taking insulin. By utilizing this tool, clinicians can more accurately assess patients’ mortality risk and implement targeted interventions for high-risk individuals, potentially reducing the risk of premature death in patients with DR. Future research should focus on prospective and interventional studies to further validate and enhance the accuracy and reliability of this nomogram.

## Data Availability

The datasets presented in this article are not readily available because The data in our study are publicly available online from the NHANES https://www.cdc.gov/nchs/nhanes/index.htm. Requests to access the datasets should be directed to https://www.cdc.gov/nchs/nhanes/index.htm.
