# Supplementary material for: Construction of a nomogram for predicting the risk of all-cause mortality in patients with diabetic retinopathy
Source: Front Endocrinol (Lausanne). 2025 Feb 21;16:1493984. doi: 10.3389/fendo.2025.1493984 (PMC11885145; doi:10.3389/fendo.2025.1493984)
Supplement: Supplementary file 1 [file DataSheet1.docx]

**Calculation of the C-index**

The C-index is used to measure the predictive accuracy of survival models. It evaluates how well the predicted survival times align with the actual observed data. The calculation involves the following steps:

1. **Pairwise Evaluation**: All study subjects are randomly paired. For n subjects, the total number of possible pairs is given by the combination formula:

***C(n,2)*** $\boldsymbol{=}\frac{\boldsymbol{n!}}{\boldsymbol{2!}\boldsymbol{*}\left( \boldsymbol{n-2} \right)\boldsymbol{!}}\boldsymbol{=}\frac{\boldsymbol{n}\boldsymbol{(n-1)}}{\boldsymbol{2}}$

*C(n,2) is the permutation*

1. **Exclusion of Invalid Pairs**: Certain pairs are excluded from the analysis:

Pairs where the subject with a shorter observed survival time is censored (i.e., the event of interest did not occur during the observation period).

Pairs where both subjects did not reach the event of interest (i.e., both are censored).

1. **Concordance Assessment**: For each valid pair, concordance is determined by comparing the predicted and actual outcomes:

A pair is concordant if the subject with the longer observed survival time also has a longer predicted survival time, or if the subject with a higher predicted survival probability actually survives longer than the other subject.

1. **C-index Calculation**: The C-index is calculated as the proportion of concordant pairs among the valid pairs:

***C-index***$\boldsymbol{=}\frac{\boldsymbol{Number of concordant pairs}}{\boldsymbol{Number of valid pairs}}$
