# Supplementary material for: Construction of a nomogram for predicting the risk of all-cause mortality in patients with diabetic retinopathy
Source: Front Endocrinol (Lausanne). 2025 Feb 21;16:1493984. doi: 10.3389/fendo.2025.1493984 (PMC11885145; doi:10.3389/fendo.2025.1493984)
Supplement: Supplementary file 2 [file Table1.docx]

**TABLE S1** Cox proportional hazards regression to select variables.

| Variables | Hazard ratio | 95% CI | p-value |
| --- | --- | --- | --- |
| Age | 1.05 | (1.04, 1.07) | <0.001 |
| Marital status |  |  |  |
| Married/Living with Partner | 1.00 (Ref.) | 1.00 (Ref.) |  |
| Widowed/Divorced/Separated | 1.13 | (0.85, 1.50) | 0.380 |
| Never married | 2.06 | (1.19, 3.58) | 0.010 |
| PIR | 0.91 | (0.83, 1.01) | 0.070 |
| CHF |  |  |  |
| No | 1.00 (Ref.) | 1.00 (Ref.) |  |
| Yes | 1.58 | (1.12, 2.24) | 0.009 |
| CHD |  |  |  |
| No | 1.00 (Ref.) | 1.00 (Ref.) |  |
| Yes | 1.45 | (1.04, 2.02) | 0.028 |
| Stroke |  |  |  |
| No | 1.00 (Ref.) | 1.00 (Ref.) |  |
| Yes | 1.41 | (1.01, 1.98) | 0.046 |
| Taking insulin |  |  |  |
| No | 1.00 (Ref.) | 1.00 (Ref.) |  |
| Yes | 1.31 | (1.01, 1.71) | 0.040 |
| Red blood cell | 0.83 | (0.63,1.07) | 0.160 |
| Creatinine | 1.00 | (1.00, 1.00) | <0.001 |

PIR, poverty income ratio; CHF, congestive heart failure; CHD, coronary heart disease.
